# The Role of Artificial Intelligence in the Characterization and Outcome Prediction of Prostate Cancer: A Systematic Review

**DOI:** 10.3390/tomography12050062

**Published:** 2026-04-28

**Authors:** Shahd Aljoudi, Aasiya Khan, Iman Dajani, Minatullah Al-Ani, Michael Mina, Dounia Baroudi, Sama Al-Saffar, Souha Aouadi, Tarraf Torfeh, Rabih Hammoud, Noora Al Hammadi, Mohammad S. Yousef

**Affiliations:** 1 Weill Cornell Medicine Qatar, Education City, Qatar Foundation, Al Luqta St. Ar-Rayyan, Doha P.O. Box 24144, Qatar; afk4004@qatar-med.cornell.edu (A.K.); ihd4001@qatar-med.cornell.edu (I.D.); mia4021@qatar-med.cornell.edu (M.A.-A.); mim4035@qatar-med.cornell.edu (M.M.); dob4005@qatar-med.cornell.edu (D.B.); sqa4003@qatar-med.cornell.edu (S.A.-S.); 2 Department of Radiation Oncology, National Centre for Cancer Care and Research, Hamad Medical Corporation, Doha P.O. Box 3050, Qatar; saouadi@hamad.qa (S.A.); ttorfeh@hamad.qa (T.T.); rhammoud2@hamad.qa (R.H.); nalhammadi1@hamad.qa (N.A.H.)

**Keywords:** artificial intelligence, prostate cancer, tumor characterization, outcome prediction, radiomics, machine learning

## Abstract

Background/Objectives: Prostate cancer (PCa) is the second most commonly diagnosed cancer in men globally. Radiation oncologists often find PCa tumor characterization and outcome prediction challenging. Therefore, the potential for artificial intelligence (AI) implementation in radiation oncology has increased in recent years. This systematic review aims to evaluate the efficacy of AI algorithms in characterizing PCa tumors and predicting post-therapy outcomes. Methods: A total of 2055 studies were identified through a comprehensive search across PubMed and Scopus, then exported to Covidence. Inclusion criteria focused on prospective and retrospective cohort studies as well as randomized clinical trials (RCTs) published between 2015 and 2024 that explored the implementation of AI in tumor characterization and outcome prediction of PCa. Two independent reviewers evaluated each paper, and evaluation metrics such as specificity, sensitivity, accuracy, and area under the curve (AUC) were analyzed. The Risk of Bias in Non-randomized Studies of Interventions, Version 2 (ROBINS-I V2) tool was used to assess the risk of bias (ROB). Results: Across the 19 studies analyzed, there was no significant difference in model performance between machine learning (ML) and deep learning (DL) models. AI models using multi-input strategies (e.g., radiomics with clinical markers) generally performed better than single-input models. Of the imaging modalities used for radiomic feature extraction, multiparametric MRI (mpMRI)-trained AI models consistently achieved the highest performance. Conclusions: AI displays considerable potential for integration into clinical workflows for PCa management. However, further studies utilizing larger datasets and external cohorts independent of the sample population are needed to validate clinical utility and improve model transparency for reliable implementation.

## Simple Summary

The detection and management of prostate cancer relies heavily on medical imaging techniques. However, accurately identifying tumor features and predicting outcomes remains challenging. This systematic review demonstrates how artificial intelligence can be utilized to improve tumor characterization and outcome prediction. Models that combine imaging with clinical information outperformed those using a single data source, with multiparametric MRIs showing the most consistent accuracy. These findings suggest that artificial intelligence can be used to support radiologists and clinicians in order to provide more personalized treatment and to improve outcomes.

## 1. Introduction

### 1.1. Background and Clinical Significance of Prostate Cancer

Cancer is a leading global cause of death and disability. PCa, a major urinary-system malignancy, is the second most common cancer in men worldwide [1]. According to the 2022 Global Cancer Statistics Report by the WHO’s International Agency for Research on Cancer (IARC), there were 1,466,718 new cases worldwide, making it the fourth most diagnosed cancer after lung, breast, and colorectal cancers and the most frequently diagnosed non-skin cancer among men [2]. In Qatar, PCa is the second most diagnosed cancer in men, accounting for 10.2% of cases [3].

PCa’s clinical challenges arise from its heterogeneity and hard-to-characterize presentation. Many cases are indolent and asymptomatic. Therefore, PSA elevation, often used for detection, can reflect benign conditions, complicating diagnosis [4]. After prostatectomy or radiotherapy, 20–40% of patients show biochemical recurrence by rising PSA, though only some progress to clinically significant disease needing intervention [5]. Radiotherapy can cause bowel and urinary side effects (e.g., hematochezia, voiding difficulty, nocturia) [6]. Accurate detection and classification depend on detailed histopathological assessment of cellular and architectural features [4,7,8,9]. Given these diagnostic and prognostic limits, AI models offer a promising means to improve tumor characterization, support clinical decisions, and enable individualized treatment planning.

### 1.2. Imaging Modalities

Computed tomography (CT) and other imaging modalities are essential for PCa diagnosis, staging, and treatment planning. CT uses X-rays for cross-sectional images, primarily to detect lymph node and bone metastases and guide biopsies in staging, but is less sensitive than MRI for local tumor detection due to limited soft-tissue contrast [10,11]. AI can improve CT by reducing manual errors (e.g., positioning), enhancing low-dose image quality, and increasing diagnostic accuracy through better lesion detection and segmentation [12,13].

MRI provides high soft-tissue contrast for local prostate staging, biopsy guidance, and tumor extent assessment [10,14]. However, interpretation is time-consuming, operator-dependent, and risks missing small tumors; AI can streamline reads, shorten scan times, and improve lesion detection [15].

While conventional MRI focuses primarily on anatomical detail, mpMRI combines anatomical and functional sequences such as T2, which are diffusion-weighted and contrast-enhanced to improve localization, risk stratification, and detection of prostate tumors versus conventional MRI [16,17,18]. It is the preferred method for PCa detection and staging and helps reduce overdiagnosis of low-risk disease, especially with elevated PSA or prior negative biopsies [19,20,21]. However, mpMRI interpretation is complex, shows high inter-reader variability, and needs subspecialty training; AI can automate lesion detection and classification to reduce missed diagnoses and variability [22,23].

### 1.3. Use of AI in Radiation Oncology

Radiation oncology faces continued challenges in tumor delineation, dose optimization, and outcome prediction [24,25,26]. In comparison, AI increasingly automates image segmentation, predicts toxicity, and integrates radiomic, clinical, and molecular data to personalize and optimize radiation plans. By leveraging large datasets, AI reduces inter-observer variability, improving treatment precision and outcomes [27,28,29,30].

### 1.4. Machine Learning vs. Deep Learning

Machine learning (ML) works well on structured data using explicit feature engineering, while deep learning (DL) uses multilayer neural networks to learn hierarchical features from unstructured inputs (e.g., imaging, genomics) [31,32,33,34,35]. ML offers interpretability and efficiency; DL provides greater accuracy and scalability with enough data. Choice depends on dataset size, complexity, and required transparency [31]. In this review, ML models are algorithms that use structured inputs and engineered features (e.g., radiomics or clinical variables), including logistic regression, support vector machines, random forests, and gradient boosting (e.g., XGBoost, LightGBM).

DL models are neural network-based architectures (e.g., convolutional neural networks) that learn hierarchical feature representations directly from raw data, typically imaging or genomic sequences, in an end-to-end or semi end-to-end manner.

### 1.5. Classification, Regression, and Clustering Algorithms

AI in medical imaging and oncology mainly uses ML for classification, regression, and clustering, differing by output type, input data, and whether labels are present [36]. Classification assigns data to predefined categories (e.g., disease present vs. absent) [37]. Support vector machines (SVMs) find optimal boundaries for linear and nonlinear class separation. Neural networks (NNs) transform inputs via layered functions to handle complex, high-dimensional data [38]. Logistic regression models the probability of a binary outcome from categorical or continuous predictor variables [39]. Random forests (RFs) are ensembles of decision trees (DTs) that reduce variance and improve predictive accuracy [40].

Regression can model relationships between quantitative variables (e.g., predicting tumor size from imaging) [41]. Common methods include linear regression for linear relationships [42] and Extreme Gradient Boosting (XGBoost), a fast, scalable, gradient-boosting tree algorithm effective for structured data [43]. It is important to note that XGBoost, and algorithms like SVMs, NNs, logistic regression, and RFs, can also be applied to classification or regression tasks as needed.

### 1.6. Objective of the Systematic Review

This systematic review evaluates AI applications in prostate cancer radiation oncology, focusing on AI-assisted tumor characterization (location, grade, stage) and outcome prediction (biochemical recurrence, radiation toxicity, survival, radiation-induced fractures), and examines strengths, limitations, and future directions to improve diagnostic accuracy, treatment planning, follow-up, and personalized care.

## 2. Materials and Methods

### 2.1. Study Selection

This systematic review followed Preferred Reporting Items for Systematic Reviews and Meta-Analyses (PRISMA) guidelines and is registered in Research Registry under the Unique Identifying Number (UIN) 2081. A comprehensive search was conducted across Scopus and PubMed from inception to August 2024 (full strategies in Supplementary Section S1), plus backward/forward citation tracking and related article screening. The 2055 yielded studies were uploaded to the systematic review tool Covidence (www.covidence.org, last accessed on 21 April 2026). Inclusion criteria: (1) prospective cohorts, RCTs, or prospectively collected data analyzed retrospectively; (2) studies of AI for PCa characterization or outcome prediction; (3) published in English; (4) published between 2015 and 2024; (5) adult study populations. Exclusion criteria: (1) animal or in vitro studies; (2) non-peer-reviewed items (conference abstracts, editorials, commentaries, letters); (3) pre-2015 studies; (4) studies of characterization/outcome prediction without AI; (5) studies focusing solely on technical tasks (e.g., segmentation or registration) without clinically relevant diagnostic or outcome endpoints. Conference abstracts were excluded due to limited methods, lack of full peer review, and duplication risk; full papers were used when available. Two reviewers screened and selected studies independently, resolving disagreements by discussion or a third reviewer.

### 2.2. Data Extraction and Analysis

Data from the 19 included studies were extracted into a master table (Table 1 and Table S2, Section S2) covering: (1) study details (objective, sample size, mean age), and (2) technical details (imaging, AI task type—classification/regression/clustering/other—inputs/outputs, validation, and performance metrics such as sensitivity, specificity, accuracy, AUC, precision). Study objectives were classified as tumor characterization (e.g., Gleason scoring), outcome prediction (e.g., recurrence, radiation toxicity), or both. Although three of the studies included were by the same first author (Abdollahi; Refs. [44,45,46]), each one evaluated distinct clinical endpoints using differing analytical approaches.

We categorized input and feature paradigms as: (1) radiomics-only (hand-crafted imaging features); (2) non-radiomics (clinical, dosimetric, genomic/biomolecular data); (3) multi-input (radiomics plus one or more non-radiomic types). Whole-image CNNs without explicit feature engineering were classified as DL-based and distinct from traditional radiomics.

Radiomics denotes hand-crafted quantitative features (e.g., texture, shape, intensity) extracted from segmented images for ML input. Deep radiomics refers to features automatically learned by CNNs from images, used as inputs or within end-to-end models. End-to-end DL models map raw imaging directly to predictions without explicit intermediate feature extraction.

Two coauthors independently extracted performance metrics to assess algorithm effectiveness and reconciled discrepancies by discussion or a third reviewer. Most reported performance metrics were derived from internal validation settings (e.g., cross-validation (CV) or hold-out test sets), reflecting current practices in the literature. Where available, externally validated results are explicitly indicated. The predominance of internally validated models may limit generalizability and was considered when interpreting comparative performance across studies. Heterogeneous endpoints, modalities, algorithms, and validations precluded meta-analysis, so we used structured narrative synthesis.

### 2.3. Quality Assessment

**Figure 1:**
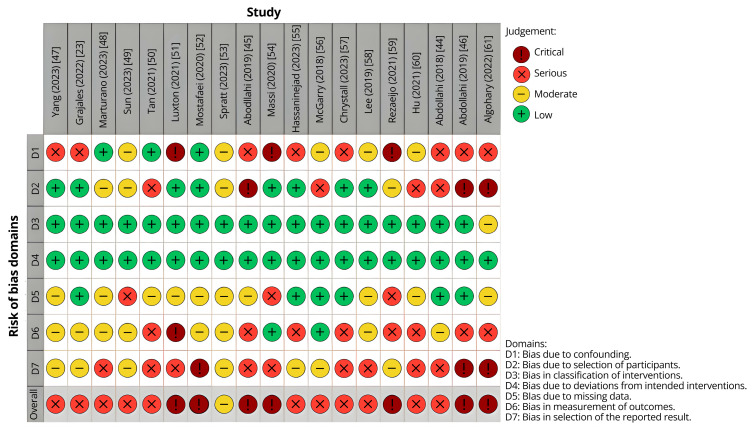
Traffic light plot summarizing the ROB for each study included in this systematic review [23,44,45,46,47,48,49,50,51,52,53,54,55,56,57,58,59,60,61].

The ROBINS-I V2 assessment tool was used to assess ROB, which is a measure of any errors in the analyzed studies that could undermine this review’s results. A comprehensive quality assessment table is provided and can be found in Table S3 (Section S3), and a traffic light plot summary is shown in Figure 1 (see Section 2 and Section 3). The overall ROB rating in the ROBINS-I V2 framework is not a simple average of all domains but is instead weighted toward the domain demonstrating the highest level of bias. Two coauthors rated each study independently and reconciled disagreements, with a third reviewer adjudicating unresolved disputes.

## 3. Results

### 3.1. Study Characteristics

**Figure 2:**
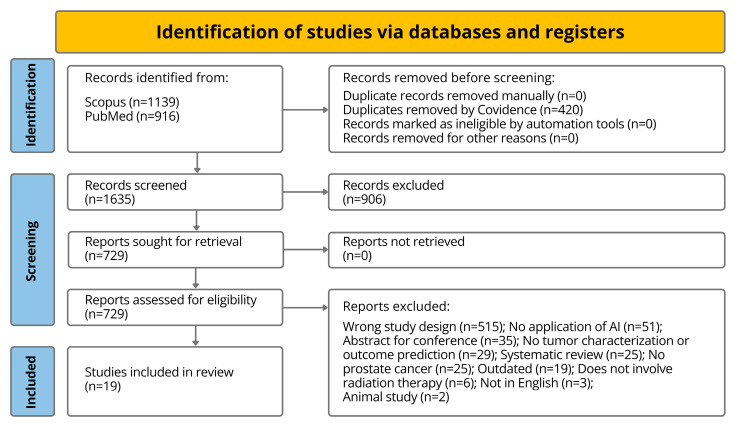
PRISMA flowchart of the number of studies in each stage of screening.

After screening for duplicates, the preliminary 2055 studies obtained were reduced to 1635 studies. Title and abstract screening left 729 studies and full-text screening yielded 19 studies for final extraction (corresponding to references [23,44,45,46,47,48,49,50,51,52,53,54,55,56,57,58,59,60,61]). Figure 2 displays a flowchart of the screening process. All included studies are consistently represented across the flowchart (Figure 2), extraction table (Table 1), and references list.

A comprehensive table detailing the subgroupings of the studies can be found in Table S4 (Section S4). This systematic review included 19 studies, primarily from two countries: the United States (seven studies) and Iran (six studies). Fewer contributions came from China (two), Canada (two), and Italy (two). Three studies analyzed data from multiple countries. Most studies were conducted recently, with the highest number published in 2023 (six studies), followed by 2022, 2021, and 2018 (three studies each). ML techniques were predominantly used (16 studies), while only three studies employed DL.

Seven studies used radiomics alone; six combined radiomics with clinical, dosimetric, or biomolecular inputs. Six studies omitted radiomics, using clinicopathological features, telomere dynamics, chromosomal instability, SNPs, or whole-image features instead.

Most studies focused on outcome prediction (12 studies), while fewer concentrated on tumor characterization (five studies). Predicted outcomes included likelihood of biochemical recurrence and radiation-induced toxicity such as GI toxicity, hemorrhage, and proctitis. Only two studies investigated both simultaneously. Prospective studies were the most common (11), while seven studies were prospective in data collection but retrospective in analysis. Only one RCT was included.

Regarding imaging modalities, CT and mpMRI were most frequently used (five studies each), followed by studies utilizing MRI alone (four studies). Other modalities such as PET imaging and in situ Raman spectroscopy were less common.

Classification algorithms were the main methodological approach, with SVM, logistic regression (five studies each), and RF (four studies) being the most frequently used. Less common classification methods included NN, k-nearest neighbors (kNN), and DTs. These classification models were used to sort data into predefined categories, such as the presence or absence of toxicity or the Gleason score of a tumor.

A small number of studies used regression algorithms, linear regression, PLS regression, preconditioned RF regression, and XGBoost (each used once). XGBoost was used as a ML model in this study. Clustering methods were rare, appearing in only one study.

### 3.2. Trends Between Tumor Characterization and Outcome Prediction Studies

#### 3.2.1. Machine Learning vs. Deep Learning Utilization

There was no clear trend indicating that ML or DL was used more/less frequently when comparing studies evaluating models’ ability to characterize tumors or predict specific outcomes. Overall, most studies, regardless of their objectives, utilized ML methods. A total of 80% of the tumor characterization studies employed ML methods [23,49,56,59], while only one study [57] employed a DL method. Similarly, for outcome prediction studies, 10 out of 12 of the studies analyzed utilized ML [44,45,47,48,50,51,52,55,58,60,62], with only two studies utilizing DL methods [53,54].

Regarding the type of algorithm family, there is no clear trend indicating a significant shift from ML to DL in recent years. In 2018–2019, all studies relied on classic ML classifiers or regressors. DL was first used in 2020 [54] and increased to two papers in 2023 [53,57]. Accordingly, the share of DL studies rose from 0 to 17%, moving from 0 in 2018–2019 to 1 in 6 during 2020–2022, and up to 33% in 2023 (two out of six studies). Therefore, while there is a slight shift to DL, the limited number of studies included in this review prevents drawing a confident conclusion that this pattern will continue.

#### 3.2.2. Relationships Between Input Type and Year of Publication

Regarding the types of inputs used as the foundation of the algorithms, there has been only a minimal shift from exclusively radiomics-based inputs to combined inputs or non-radiomic inputs in recent years. In 2018 and 2019, a total of five studies each relied solely on radiomics [44,45,46,56,58], but the number decreased to one study per year in 2021, 2022, and 2023 [55,59,61]. Additionally, one study per year from 2020 to 2024 used radiomics in combination with other features [23,47,48,49,52,60], indicating no growth in popularity of this category of input. The first study using non-radiomic inputs appeared in 2018, and only one study per year employed such input from 2020 to 2023. Overall, there was no clear increase in non-radiomic inputs or a major shift from radiomics-only to non-radiomics inputs. Given the small sample size (19 studies) and limited time span (2018–2024), no strong correlation between the publication year and the input type can be concluded.

### 3.3. Trends in Performance Between Different Subgroups

#### 3.3.1. Studies Utilizing ML vs. DL Models

Studies that used ML as opposed to DL, primarily end-to-end CNN-based models, did not consistently perform better or worse. To expand, only one of the three DL studies applied their model to tumor characterization, while four studies used ML models for tumor characterization. Therefore, an adequate comparison of ML vs. DL performance for characterization of PCa tumors is difficult. Chrystall et al. (2023) [57], who used DL for marker detection in multi-cycle cine images, reported an externally validated AUC of 0.937, sensitivity of 98.31%, and specificity of 99.87%. The highest AUC reported among the four ML studies was AUC = 0.83 (95% 0.76–0.91) [49] in detecting PCa peripheral zones. The other three ML studies reported mediocre AUC, sensitivity, and specificity scores ranging from AUC = 0.5–0.7 [47], specificity = 87–92%, and sensitivity = 83–90% (95% CI: 0.71–0.88) [23]. Rezaeijo et al. (2021) [59], the final ML study, used none of these three analyses metrics so the performance of their model cannot be compared to the others. Ultimately, the highest performing ML and DL models for tumor characterization are comparable; however, the average ML model performs worse in tumor characterization compared to DL. Conversely, comparing ML and DL models utilized for outcome prediction was more difficult. The highest ML studies reached AUCs around 0.89–0.93 (e.g., 0.894 (95% CI: 0.71–0.88) in [50] and 0.93 (95% CI: 0.70–0.87) in [60]). The only DL paper for outcome prediction did not provide AUC, accuracy, specificity, or sensitivity figures and focused on genomics [53], offering no direct path of comparison.

#### 3.3.2. Studies Using Radiomics vs. Non-Radiomics vs. Combined Inputs

In addition, there was a notable difference amongst models that used radiomics-only inputs, non-radiomics-only inputs (e.g., SNPs, telomere length), or utilized a multi-input approach. Radiomics-only papers clustered in the mid 0.7 AUC range. For instance, the study by Sun et al. (2023) [49] reported validation AUCs of 0.79 for B-mode and 0.78 for CEUS when each modality’s radiomics were used alone. GI toxicity prediction reached 0.71 and dipped after clinical variables were added (combined AUC = 0.65) [55]. Studies that used only non-radiomics inputs (e.g., clinical features of SNPs) performed slightly better than the radiomics-only inputs, often achieving AUC scores of >0.85. For instance, Tan et al.’s (2021) [50] large clinical features dataset achieved AUCs of 0.83–0.89 across three ML classifiers for recurrence prediction. In addition, Chrystall et al. (2023) [57], which relied on end-to-end deep learning models operating on whole image CNN features, achieved an externally validated AUC of 0.93—well above the 0.85 threshold. The best AUC and accuracy scores were observed for multi-input models utilizing at least two inputs. Pairing mpMRI radiomics with PSA raised the AUC to 0.93 compared to 0.92 for radiomics alone and 0.74 for PSA alone [60]. Similarly, supplementing PET radiomics with clinical variables improved the baseline from 0.73 to 0.78 [48]. Moreover, combining MRI radiomics with dosimetry improved kNN accuracy, achieving an AUC of 0.86 [58].

The main outlier to this trend was Hassaninejad’s study focusing on toxicity, where radiomics-only scores were slightly higher than the combined model [55]. In summary, radiomics alone achieved good but not outstanding results (AUC 0.7–0.8), while non-radiomic and multi-input models generally performed better, with scores up to 0.93 AUC.

#### 3.3.3. Studies Using Classification vs. Regression vs. Clustering vs. Other Algorithms

Regarding trends between studies using different algorithm methods, this systematic review found that the 16 classification studies reported a wide range of AUC values, with some strong papers reporting AUCs near 0.9. Tan et al.’s (2021) [50] NB model for biochemical recurrence achieved an AUC of 0.894, while Yang et al.’s (2023) [47] rectal toxicity LightGBM scored an AUC of 0.741 (95% CI: 0.719–0.762) on the test set. For the three regression studies, R^2^ values were reported. Since these values are on a different scale to AUC, they cannot be directly compared with classification studies. Luxton et al. (2021) [51] used XGBoost regression to predict post-radiotherapy telomere metrics, achieving R^2^ = 0.88 on hold-out patients. However, its linear regression performed much worse (R^2^ < 0.55). In the recorded clustering study [59], hierarchical clustering of mpMRI radiomic features was applied for dose-painting feasibility. There was no reported AUC or R^2^ value; the study only stated that dosimetric outcomes were not significantly different between plans.

### 3.4. Impact of Imaging Modality on AI Performance

AI models were developed using various imaging modalities, each yielding differing performance outcomes. mpMRI consistently demonstrated the highest diagnostic accuracy across studies. For instance, McGarry et al. (2018) [56] used mpMRI combining T2WI and DCE sequences to classify PCa lesions by grade. The resulting radiomics model achieved high diagnostic performance, with AUC values between 0.82 and 0.91, as well as strong agreement between radiomic heatmaps and pathologist-identified tumor regions. Similarly, Hu et al. (2021) [60] combined T2WI and multiple DWI sequences, reporting AUCs of 0.85 for each sequence. When these radiomic features were integrated with clinical variables (such as total PSA, free PSA, and the free/total PSA ratio), the model’s performance improved further, achieving an AUC of 0.93 and a sensitivity of 0.94 [60]. Together, these findings highlight the strong diagnostic potential of mpMRI-based radiomics in identifying PCa. Furthermore, ultrasound-based models also showed strong diagnostic capability. Sun et al. (2023) [49] reported a validation AUC of 0.83 (95% CI: 0.76–0.91) for detecting peripheral-zone PCa when combining B-mode and CEUS radiomics, underscoring the diagnostic value of multi-parametric ultrasound inputs. Overall, while ultrasound-based models show promising results, mpMRI remains the more accurate and reliable imaging modality for PCa detection, consistently achieving higher diagnostic performance across studies.

In contrast, CT-based radiomic models generally demonstrated moderate predictive accuracy. For example, one study used planning CT to predict RT-induced toxicity and reported test AUC values between 0.6 and 0.7 [47]. Specifically, the model predicting radiation-induced hemorrhage using combined radiomic and dosimetric features achieved a test AUC of 0.741 (95% CI: 0.719–0.762) [47]. Another study used CT in combination with MRI for bladder wall segmentation [46]. Although radiomic features were extracted from MRI, the inclusion of CT improved precision in segmentation. AUCs for the classification model detecting patients who developed grade ≥2 urinary toxicity ranged from 0.62 to 0.75, highlighting the potential utility of CT-MRI hybrid approaches for toxicity prediction. Similar to CT, PET-based models demonstrated intermediate performance. Marturano et al. (2023) [48] developed a radiomics model using fluoromethylcholine PET/CT to predict biochemical recurrence, achieving an AUC of 0.78 when combined with clinical data—an improvement over the clinical-only model which had an AUC value of 0.73.

Overall, these results suggest that MRI, especially mpMRI and multi-sequence ultrasound, consistently supported higher discriminative performance in lesion detection and characterization. CT and PET, while more often used in predicting outcomes like biochemical recurrence, generally demonstrated moderate accuracy, emphasizing how the choice in imaging modality heavily impacts the performance of AI models for PCa.

### 3.5. Impact of Image Sequence Choice on AI Performance

Within a given imaging modality, differences in imaging sequences or techniques further influenced AI model results. For instance, a study conducted by Abdollahi et al. (2018) [44] compared radiomic models using various MRI sequences for prognostic assessment. Models built on advanced DWI outperformed those using standard sequences. In that study, a “zoomed” high b-value DWI (b = 2000 s/mm^2^) yielded the highest AUC of 0.89 for classifying treatment response, whereas the conventional axial T2W MRI and full-field DWI sequences achieved slightly lower AUC values, around 0.77–0.85 [44]. For example, the radiomics model using zoomed DWI (b = 1500) achieved an AUC of 0.87 with 94% specificity, whereas the T2W model had an AUC of 0.85 [44]. This trend underscores that higher-resolution or functional MRI sequences can enhance model performance in predicting PCa outcomes.

Similarly, in a study utilizing ultrasounds as the imaging modality, the use of contrast-enhanced imaging improved diagnostic accuracy. This study directly compared radiomics from conventional B-mode ultrasound against CEUS in a peripheral-zone tumor detection task [49]. The combined B-mode + CEUS radiomic model had greater accuracy, evident in the AUC values of 0.83 in the validation phase, compared to models using either CEUS or B-mode alone, which had AUC values of 0.78 and 0.79, respectively [49]. By utilizing complementary information from both sequences, the combined approach improved sensitivity (82%) and specificity (74%) for cancer detection (although these values were in the validation phase). Comparatively, the B-mode ultrasound alone showed a sensitivity of 67% while the CEUS model alone demonstrated a sensitivity of 76%. Similarly, the combined model enhanced specificity to 74%, achieving a higher specificity than the CEUS alone (65%), but slightly underperforming compared to the B-mode ultrasound alone (78%) [49]. Across studies, comparisons within the same imaging modality highlight that incorporating advanced imaging sequences (e.g., DCE-MRI, high b-value DWI, or CEUS) generally lead to better AI model performance than using a single conventional sequence alone. However, the magnitude of improvement varied by study, and some single-sequence models (e.g., T2W MRI radiomics) still achieved high accuracy on their own with an AUC of 0.85 [60]. As a general trend, tailoring the imaging protocol, either by adding contrast or increasing diffusion weighting, was associated with substantial gains in predictive performance in the reviewed studies.

### 3.6. Risk of Bias Analysis

The ROBINS-I V2 assessment (Figure 1) demonstrated that a substantial proportion of included studies were judged to have serious-to-critical overall risk of bias (ROB), consistent with the ROBINS-I framework in which the overall rating is determined by the most severely biased domain rather than an aggregate across domains. Domain-specific evaluation revealed that bias was not uniformly distributed, but instead concentrated in key methodological areas.

Bias due to confounding (D1) was a predominant contributor to elevated ROB. Several studies (e.g., Yang 2023 [47]; Grajales 2022 [23]) were conducted using retrospective, single-institution cohorts without adequate adjustment for clinically relevant covariates such as tumor stage, prior treatments, or comorbidities. This limitation has been widely recognized in AI-based prognostic modeling, where inadequate control of confounders can substantially distort model performance and clinical interpretability [63,64].

Selection bias (D2) was also frequently observed, particularly in studies relying on non-random or convenience sampling of imaging datasets (e.g., McGarry 2018 [56]; Hu 2021 [60]). Restriction to high-quality or pre-selected imaging cohorts may artificially inflate predictive accuracy and limit generalizability, a concern consistently highlighted in systematic evaluations of machine learning models [64].

Bias in classification of interventions (D3) was generally low across studies, reflecting the objective nature of imaging-derived inputs and clearly defined treatment variables.

Bias due to deviations from intended interventions (D4) was minimal, as most included studies were observational and did not involve prospective allocation or protocol deviations.

Bias due to missing data (D5) was inconsistently addressed. Several studies (e.g., Tan 2021 [50]; Massi 2020 [54]) did not clearly report methods for handling incomplete imaging or clinical data, raising concerns regarding potential bias introduced through complete-case analyses or unreported imputation strategies. Inadequate reporting of missing data handling remains a recognized limitation in AI model development studies [63].

Measurement bias (D6) represented another major contributor to elevated ROB. In multiple studies (e.g., Marturano 2023 [48]; Hassaninejad 2023 [55]), outcomes were derived from surrogate endpoints or model-generated classifications without consistent validation against standardized clinical reference standards. Additionally, lack of blinding in outcome assessment and heterogeneity in imaging acquisition protocols contributed to variability in measurement. These issues are well documented in the literature, where inconsistent outcome definitions and non-standardized pipelines reduce reproducibility and reliability [64,65].

Bias in selection of the reported result (D7) was frequently identified, particularly in studies reporting selectively favorable performance metrics without prespecified analysis plans (e.g., Luxton 2021 [51]; Abdollahi 2019 [46]). This includes omission of negative findings, lack of external validation, and preferential reporting of internally optimized model outputs. Selective reporting bias has been shown to be strongly associated with overestimated model performance in prediction modeling studies [65].

Importantly, while many studies demonstrated low-to-moderate risk within individual domains, the presence of at least one domain with serious or critical bias resulted in high overall ROB classification. This pattern aligns with prior systematic evaluations of AI applications in oncology and imaging, which consistently identify retrospective de-signs, heterogeneous datasets, and insufficient pre-specification as major contributors to bias [63,64].

The observed biases reflect both study design limitations and intrinsic challenges of AI-based radiotherapy research, including high-dimensional imaging features, algorithmic opacity, and variability in clinical implementation. Although these limitations reduce the feasibility of quantitative meta-analysis, the consistency of findings across studies and the use of a structured narrative synthesis support the robustness of the overall conclusions.

## 4. Discussion

### 4.1. Main Takeaways and Conclusions

ML was used more often than DL with no clear superiority. Multimodal/combined-input models performed best—mpMRI plus clinical variables achieved the highest AUCs (up to 0.93) [60]. CT-based models showed the lowest accuracy (AUC~0.6–0.75) [48,49], while ultrasound and PET achieved moderate AUCs (~0.73–0.83) [47]. Using advanced or multiple sequences within a modality generally improved accuracy, and classification algorithms were the most commonly used and displayed strong performance.

### 4.2. Explaining Trends Observed in Section 3

MRI radiomics outperformed CT radiomics, achieving the highest AUCs [47,56,60], likely because MRI’s superior soft-tissue contrast and resolution provides higher-quality, more comprehensive input for AI training and tumor characterization [66,67].

Not only does imaging modality influence AI model performance, but the type of sequence used within each modality plays a role. Advanced sequences (e.g., CEUS) capture physiological details (microvasculature/perfusion via microbubbles) that standard sequences like basic B-mode ultrasound might miss, improving AI discrimination [68,69]. Combining complementary sequences further boosts accuracy, but advanced/multi-sequence protocols are costlier and more time-consuming; for real-world deployment, models should preferably use standard sequences routinely acquired for treatment.

### 4.3. Comparing the Findings of This Systematic Review to the Literature

#### 4.3.1. No Difference in ML vs. DL Performance

We found no consistent performance difference between ML and DL for PCa; both achieved similar AUC ranges depending on inputs and tasks, consistent with Sushentsev et al. (DL AUC 0.8–0.9; ML AUC 0.75–0.88) [70]. Model success appears driven more by data quality and feature selection than by ML vs. DL. Olabanjo et al. (2023) reviewed 77 AI–PCa studies and found no consistent DL superiority: DL (CNNs/U-Nets) AUCs ~0.84–0.99 and traditional ML (SVM, RF) AUCs ~0.81–0.94 across detection, classification, and Gleason grading tasks [71], supporting our finding of no systematic ML vs. DL performance difference.

#### 4.3.2. No Trend Concerning a Shift from ML to DL in Recent Years

Sushentsev et al. (2022) found no temporal shift from ML to DL in MRI-based PCa studies; both remained well represented [70]. Olabanjo et al. (2023) reported increased DL use (especially CNNs and transfer learning), which also dominated diagnostics in our review, but traditional ML (SVM, RF, ensembles) remains prevalent and effective [71]. Thus, DL use has risen alongside, not replaced, ML.

#### 4.3.3. AI Models Utilizing Combined Inputs Outperform Radiomics-Only Models

Mylona et al. (2025) found ADC radiomics alone best (AUC up to 0.74), and that combining T2W+ADC did not improve performance [72]. By contrast, Zhong et al. (2025) reported that adding clinical variables to MRI radiomics improved prediction of biochemical recurrence-free survival, while adding hypoxia gene signatures did not [73]. Ferro et al. (2022) showed mpMRI + clinical models reached AUCs up to 0.94, outperforming single-input models [74]. Thus, combined models can improve performance, but benefits depend on the specific inputs.

#### 4.3.4. mpMRI-Based Models Provide the Highest Diagnostic Accuracy Compared with Other Imaging Modalities

Zhao et al. (2022) found PSMA PET/CT (AUC 0.91) outperformed mpMRI (AUC 0.84) [75], contradicting our findings. However, Dong et al. (2025) and Yana et al. (2025) report that mpMRI outperforms TRUS/PET and mpMRI-based AI AUCs up to 0.93, respectively [76,77]. Overall, most literature supports mpMRI as the highest-accuracy modality.

#### 4.3.5. CT-Based Models Exhibit the Lowest Diagnostic Accuracy Compared with Other Imaging Modalities

The literature supports that CT-based models generally underperform versus other modalities for grading, risk stratification, and toxicity prediction [78,79], though isolated CT results can be higher in specific contexts (e.g., lymph-node detection or small internal cohorts) [80,81]. For example, Ching et al. reported a CT radiomics AUC of 0.795 for 5-year PFS in high-risk PCa [78], and synthetic cone beam CT-based (CBCT) delta-radiomics showed AUCs ~0.64–0.74 for urinary toxicity [79], still below typical mpMRI performance. Overall, CT/CBCT models do not match mpMRI accuracy. Bosetti et al. (2020) [80] reported a CBCT radiomics AUC of 0.83 for risk group, stage, Gleason, and PSA prediction, indicating that CT/CBCT can perform well in specific tasks, but generally remains less remarkable across most applications.

### 4.4. The Black Box Phenomenon

**Figure 3:**
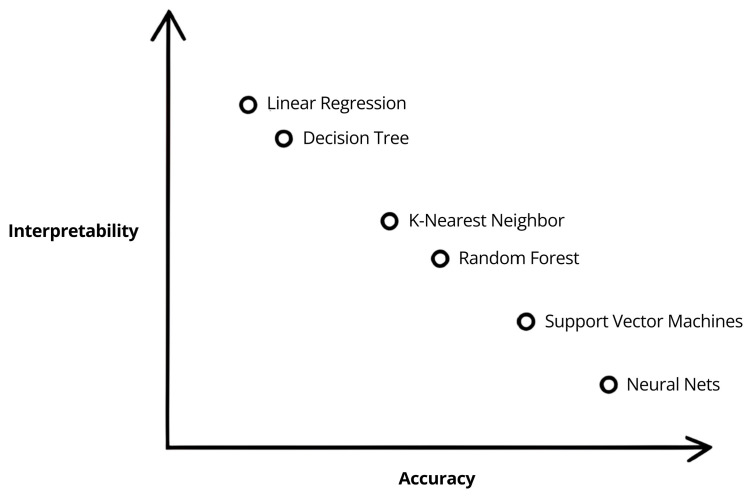
Reference image adapted from Gulum et al. (2021) illustrating which models are typically considered “black box” versus interpretable AI, and the trade-off between interpretability and accuracy [82].

Deep learning’s “black box” nature limits interpretability and hampers error correction and bias detection, posing clinical risk. By contrast, interpretable methods (e.g., logistic regression with LASSO) can reveal key features—Marturano et al. reported commonly selected radiomic and clinical predictors (Gleason, PSA), enhancing clinician trust [48]. Spratt et al. (2023) used a multimodal DL model to predict benefits from short-term ADT+RT with strong external validation, but its internal decision process was uninterpretable and lacked explainability methods (e.g., attention maps, Shapley Additive Explanations (SHAP)), making it a clinically useful but opaque “black box” [53]. DL models in medical imaging are often accurate but opaque; explainability is limited unless techniques like SHAP, local interpretable model-agnostic explanations (LIME), saliency/heat maps, and/or Gradient-weighted Class Activation Mapping (Grad-CAM) are applied [82]. Figure 3 graphically demonstrates the relationship between accuracy and interpretability of different models. As AI integrates into care, clinician understanding of model reasoning becomes essential—blind reliance risks suboptimal or harmful decisions if models are misused, biased, or applied beyond validation.

Although it may seem unimportant for clinicians to understand the AI models they utilize for diagnosis and treatment, it may rise in importance as AI becomes further integrated into healthcare. In situations where AI influences critical decisions regarding patient care, it is essential for physicians to comprehend how the model generates outputs. Blindly relying on AI may lead to suboptimal or harmful outcomes, especially if the model is used inappropriately, exhibits bias, or operates beyond its validated scope.

### 4.5. Strengths of This Systematic Review

This systematic review synthesizes 19 AI studies on PCa tumor characterization and outcome prediction, categorizing by model type (ML vs. DL) and input modality (radiomics-only, non-radiomics, multi-input), allowing for a nuanced understanding of performance trends across applications [23,48,60]. Inclusion of mpMRI, CT, PET, ultrasound, and Raman spectroscopy highlights modality-specific results, with notably superior mpMRI radiomics accuracy [49,56,60]. We included diverse research designs (prospective cohorts, retrospectives, one RCT) to balance efficacy and real-world performance [53,59]. Heterogeneity was treated as a strength—hence narrative synthesis rather than misleading meta-analysis. The RCT (Rezaeijo 2021) showed AI-guided mpMRI dose-painting improved tumor control (92% vs. 70%) without increased toxicity [59]. Distinguishing internal from external validation enabled assessment of generalizability. Furthermore, the identification of trends, such as the rise in DL studies in recent years and the shift from single-input radiomics models to multi-input frameworks, reflects how the field is evolving in complexity and clinical relevance [47,57]. Emphasizing prospective studies increases clinical relevance: predefined endpoints and protocols improve consistency, reduce selection bias, and provide more credible estimates of real-world diagnostic and prognostic performance.

### 4.6. Limitations of This Systematic Review

Limitations arise from included studies: external validation was limited and sample sizes were modest, reducing generalizability [23,49,60]. The sole RCT was a small pilot (n = 40) [59]. Prospective cohorts mostly used internal test sets without multi-institutional validation [48,49], while retrospective studies, though useful for external assessment, depend on historical data and may not reflect prospective implementation. The scarcity of multicenter cohorts and predominance of internally validated models weaken external robustness. Most studies used classification (16/19), limiting insights into regression (n = 3) and clustering (n = 1) approaches [51,56,59]. DL was underrepresented (3/19), hindering ML vs. DL comparisons [53,54,57]. Algorithm use was concentrated (SVM n = 5, logistic n = 8, RF n = 4), so findings mainly reflect popular methods rather than the full range of AI tools for PCa. Publication bias likely favored positive/high-performing studies, underscoring the need for standardized methods, larger datasets, and prospective multi-institutional validation. By not searching IEEE Xplore, Embase, or Web of Science, conference-only or early DL studies may have been missed; the exclusion of conference abstracts/proceedings (see Section 2) is a noted limitation. Observed domain biases (Figure 1) mainly limit meta-analysis feasibility due to heterogeneity; using a narrative synthesis reduces their impact on the review’s conclusions.

## 5. Conclusions

### 5.1. Summary of Findings

AI shows promise for PCa tumor characterization and outcome prediction, especially with multi-input models. Radiomics-only studies had moderate AUCs (~0.70–0.80), while radiomics combined with clinical/dosimetric data reached AUCs up to 0.93 [48,52,60]. Clinical/genetic-only models also achieved high AUCs for outcome prediction [50,53,58]. No consistent ML vs. DL advantage was found (DL studies were few). mpMRI-based radiomics (particularly combined with clinical data) consistently outperformed CT/PET radiomics [47,56,60], and advanced/multi-sequence protocols (e.g., T2W + DWI, B-mode + CEUS) improved performance [44,49]. Overall, AI success depends on data diversity, input integration, and imaging quality.

### 5.2. Impact of This Systematic Review

Organizing studies by input type, modality, algorithm, and endpoint provides a structured framework demonstrating where AI adds most value in PCa care. The review highlights the benefit of hybrid multimodal models for improved diagnostic and prognostic accuracy, offers comparative benchmarks for future research, and supports AI as an augmentative tool for personalized radiation oncology rather than a replacement for clinical expertise.

### 5.3. Recommendations for Future Research

Future work should apply this framework to other RT-driven cancers (breast, lung, brain, head and neck, GI) and further explore AI for segmentation, dose prediction, adaptive RT, and toxicity mitigation. Prospective multicenter trials, particularly RCTs, are urgently needed to validate models in real-world, heterogeneous populations [53,59]. Reporting standards must include confidence intervals, specify validation methods, and publish both positive and negative results to reduce bias and boost reproducibility. Finally, as DL use grows, comparative studies evaluating its added value over traditional ML will be essential for clinical integration.

## Figures and Tables

**Table 1:** Extracted data summary table (The full detailed Tables are provided in the Supplementary Section).

Study	Design	N	Population	Imaging Modality	AI Method	Input Features	Task	Validation Strategy	External Validation	Performance Metrics	Code/Data Shared?
Yang et al. (2023) [47]	Retrospective	183	PCa RT patients	CT	ML (SVM, LightGBM, NN)	Radiomics + dosimetric	Outcome prediction	Repeated k-fold CV + bootstrapping	No	AUC: 0.55–0.74	Yes
Grajales et al. (2022) [23]	Prospective pilot	18	PCa patients	mpMRI + Raman	ML (SVM)	Biomolecular + radiomics	Tumor characterization	Leave-one-out CV	No	Sensitivity: 83–90%	NR
Marturano et al. (2023) [48]	Prospective	74	PCa patients	PET/CT	ML (LASSO)	Radiomics + clinical	Outcome prediction	Hold-out	No	AUC: 0.78	NR
Sun et al. (2023) [49]	Prospective	166	PCa patients	TRUS + CEUS	ML (LASSO)	Radiomics + clinical	Tumor characterization	Five-fold CV	No	AUC: 0.79–0.89	NR
Tan et al. (2021) [50]	Retrospective	1130	PCa patients	N/A	ML (NB, RF, SVM)	Clinical	Outcome prediction	Train/test split	No	AUC: 0.83–0.89	NR
Luxton et al. (2021) [51]	Prospective	15	PCa patients	IMRT-related	ML (XGBoost)	Telomere + genomic	Outcome prediction	LOOCV + five-fold CV	No	R^2^ = 0.88	Yes
Mostafaei et al. (2020) [52]	Prospective	64	PCa patients	CT	ML (RF, NN, SVM)	Radiomics + clinical + dosimetric	Outcome prediction	Repeated five-fold CV	No	AUC: 0.65–0.77	NR
Spratt et al. (2023) [53]	Retrospective (RCT data)	2024 + 1594	PCa patients	Imaging + clinical	DL (ResNet-50)	Whole image + clinical	Outcome prediction	External cohort validation	Yes	HR = 0.64	Yes
Abdollahi et al. (2019) [45]	Prospective	33	PCa patients	MRI	ML (multiple classifiers)	Radiomics	Tumor characterization + outcome	Ten-fold CV	No	AUC: 0.67–0.74	NR
Massi et al. (2020) [54]	Retrospective	1401	PCa patients	Genomic	DL (Autoencoder)	SNPs	Outcome prediction	Repeated subsampling	No	Qualitative SNP validation	NR
Hassaninejad et al. (2023) [55]	Prospective	70	PCa patients	MRI	ML (RF, DT, LR, kNN)	Radiomics + dosimetric	Outcome prediction	Five-fold CV	No	AUC: 0.86	NR
McGarry et al. (2018) [56]	Prospective	39	PCa patients	mpMRI	ML (PLS)	Radiomics	Tumor characterization	Multi-step validation	No	Dice: 94.5%, R = 0.99	NR
Chrystall et al. (2023) [57]	Retrospective	29	PCa patients	SBRT imaging	DL (CNN)	Whole image	Tumor characterization	Independent test set	Yes	AUC: 0.93	NR
Lee et al. (2019) [58]	Retrospective	324	PCa patients	Genomic	ML (RF regression)	SNPs	Outcome prediction	CV + hold-out	Partial	AUC: 0.67–0.70	NR
Rezaeijo et al. (2021) [59]	RCT	120	PCa patients	mpMRI	ML (clustering)	Radiomics	Tumor characterization	Statistical validation	No	Tumor control probability: 91.8% vs. 70.5%	NR
Hu et al. (2021) [60]	Prospective	136	PCa patients	mpMRI	ML (LASSO)	Radiomics + clinical	Outcome prediction	Hold-out	No	AUC: 0.92–0.93	NR
Abdollahi et al. (2018) [44]	Prospective	33	PCa patients	MRI	ML (LR)	Radiomics	Outcome prediction	Five-fold CV	No	AUC: 0.56–0.58	NR
Abdollahi et al. (2019) [46]	Prospective	33	PCa patients	MRI	ML (LR)	Radiomics	Outcome prediction	Internal validation	No	AUC: 0.65	NR
Algohary et al. (2022) [61]	Retrospective	25	PCa patients	mpMRI	ML (LR)	Radiomics	Tumor characterization + outcome	LOOCV	No	Volume metrics	NR

## Data Availability

No new data were created or analyzed in this study. All data supporting the findings of this review are derived from previously published studies cited within the manuscript.

## Supplementary Materials

The following supporting information can be downloaded at: https://www.mdpi.com/article/10.3390/tomography12050062/s1, PRISMA 2020 Checklist [83]; Section S1. Full Search Strategy; Section S2. Data extraction (Table S2. Data extraction of the final 19 eligible studies); Section S3. The Risk Of Bias In Non-Randomized Studies of Interventions, Version 2 (ROBINS-I V2) (Table S3. Raw ROB table (using ROBINS-I V2)); Section S4. Study Categorization (Table S4. Categorization table of the 19 eligible studies).

## Abbreviations

The following abbreviations are used in this manuscript:

PCa	Prostate cancer
AI	Artificial Intelligence
AUC	Area under the curve
ROBINS-I V2	Risk of Bias in Non-randomized Studies of Interventions, Version 2
ROB	Risk of Bias
MRI	Magnetic Resonance Imaging
mpMRI	Multiparametric MRI
PSA	Prostate specific antigen
WHO	World Health Organization
CT	Computed Tomography
ML	Machine learning
DL	Deep learning
SVMs	Support Vector Machines
NNs	Neural Networks
NR	Not Reported
GI	Gastrointestinal
LightGBM	Light gradient boosting machine
CV	Cross validation
IMRT	Intensity-Modulated Radiation Therapy
ISUP GG	International Society of Urological Pathology Grade Group
PET	Positron Emission Topography
LASSO	Least Absolute Shrinkage and Selection Operator
NB	Naive Bayes
DT	Decision Tree
SNPs	Single-nucleotide polymorphisms
RCT	Randomized Controlled Trial
RT	Radiation Therapy
kNNs	K-nearest neighbors
RF	Random Forest
PLSs	Partial Least Squares
XGBoost	Extreme Gradient Boosting
ADBO	Adaptive Boosting
B-mode	Brightness-mode
CEUS	Contrast-Enhanced Ultrasound
T2W	T2-weighted
T2W1	T2-weighted imaging
DCE	Dynamic Contrast-Enhance
DWI	Diffusion-Weighted Imaging
ADC	Apparent Diffusion Coefficient
D	Domain
PSMA	Prostate-Specific Membrane Antigen
TRUS	Transrectal Ultrasound
SHAP	Shapley Additive Explanations
LIME	Local Interpretable Model-agnostic Explanation
Grad CAM	Gradient-weighted Class Activation Mapping
