# Supplementary material for: The Role of Artificial Intelligence in the Characterization and Outcome Prediction of Prostate Cancer: A Systematic Review
Source: Tomography. 2026 Apr 28;12(5):62. doi: 10.3390/tomography12050062 (PMC13210923; doi:10.3390/tomography12050062)
Supplement: Supplementary file 1 [file tomography-12-00062-s001.zip › tomography-4163080-supplementary/tomography-4163080-supplementary.pdf]

Section S1. Full Search Strategy

PubMed (MEDLINE)

Date: July 25, 2024.
Search strategy: (RT OR radiation therapy OR radiotherapy) AND (AI OR artificial intelligence OR deep learning OR machine learning OR radiomics) AND (prostate cancer*).
Filters applied: English language, publication date from 2015–2026.
Study Design: No restrictions on study design were applied during the search; eligible study types were selected during the screening process according to predefined inclusion criteria.

Scopus

Date: August 12, 2024.
Search strategy: TITLE-ABS-KEY ((RT OR radiation therapy OR radiotherapy) AND (AI OR artificial intelligence OR deep learning OR machine learning OR radiomics) AND (prostate cancer*)
Filters applied: English language, publication date from 2015–2026.
Study Design: No restrictions on study design were applied during the search; eligible study types. were selected during the screening process according to predefined inclusion criteria.

Section S2. Data Extraction

Table S2. Data extraction of the final 19 eligible studies.

**Study Reference**	**Study Type**<br><br>**Country**	**Outcome prediction, tumor characterization or both**	**N (total)**<br><br>**Age: Mean ±SE <u>or</u> Median (IQR) <u>or</u> Median (Range) (years)**	**Patient treatment details**	**Algorithmic input**	**ML or DL**	**Classification/ regression/ clustering/other method**	**Feature selection**	**Validation method**	**Results & clinical outcomes**
Yang, Z., et al (2023) [47]	Prospective data collection; retrospective analysis of data<br><br>United Kingdom	Outcome prediction<br><br>Predict radiation-induced toxicity endpoints: haemorrhage, proctitis, and Gastrointestinal (GI) toxicity	N = 183<br>Age: NA	Intensity-Modulated Radiation Therapy (IMRT):<br><br>N = 107 patients; 75 Gy in 37 fractions<br>N = 76 patients; 60 Gy in 20 fractions.	Dosimetric features & radiomic features of rectal wall extracted from CT	ML	**Classification:** SVM, Light gradient boosting machine (LightGBM), and NN	Principal component analysis, Variance threshold, Correlation threshold<br>RF	Repeated k-Fold Cross-Validation, Bootstrapping	AUC Values:<br>Proctitis prediction: 0.549<br>Hemorrhage prediction: 0.741<br>GI toxicity prediction: 0.669<br>Combined model: 0.747
Grajales, D., et al (2022) [23]	Prospective pilot clinical study<br><br>Canada	Tumor characterization<br><br>Predict International Society of Urological Pathology Grade Group (ISUP GG) of lesion	N = 18<br>Median (range): 68 years (60-74 years)	High-dose-rate brachytherapy	Biomolecule features from in-situ Raman Spectroscopy combined with radiomic features from mpMRI	ML	**Classification:** SVM	Variance Threshold, Correlation-based Feature Selection<br>Least Absolute Shrinkage and Selection Operator (LASSO) regression	Leave-One-Out Cross-validation	**ISUP GG > 1:**<br>Sensitivity: 90%<br>**ISUP GG ≥ 1**<br>Sensitivity: 83%<br>**High grade**<br>Sensitivity: 89%

Marturano, F., et al. (2023) [48]	Prospective observational study<br><br>Italy	Outcome prediction<br><br>Predict biochemical recurrence in intermediate and high risk Pca patients	N = 74<br>Median age = 73<br>Range = 43-86	39 patients (53%) treated w/ radial prostatectomy<br>35 (47%) patients treated w/ definitive radiation therapy (RT) (unspecified)	Positron Emission Topography (PET)/CT Radiomics & clinical data (Gleason score & PSA)	ML	**Classification:** LASSO logistic regression	1. Correlation-based redundancy removal<br>2. Model-based feature selection with LASSO via SelectFromModel	30-repeated hold-out validation	**PSA + Gleason score:**<br>AUC = 0.73.<br>**Radiomics + PSA + Gleason:**<br>AUC = 0.78
Sun, Y., et al. (2023) [49]	Prospective cohort study<br><br>China	Tumor characterization<br><br>Detection of Pca lesion in peripheral zone	N = 166<br>Mean age = 73 ± 8.8 years<br>Age range = 47-83	NR	Brightness-mode (B-mode) transrectal ultrasound (TRUS) and Contrast-Enhanced Ultrasound (CEUS) radiomics + risk factors (age, serum total PSA, free PSA, free/total PSA, prostate volume, and PSA density	ML	**Classification:** LASSO logistic regression	L1-based LASSO	5-fold cross-validation	AUC Values:<br>B-mode radiomics: 0.79<br>CEUS radiomics: 0.78<br>B-mode CEUS combined model: 0.83<br>Risk factor-radiomics combined model: 0.89
Tan, Y., et al. (2021) [50]	Retrospective cohort study using a prospective registry<br><br>Singapore	Outcome prediction<br><br>Predict biochemical recurrence after radical prostatectomy	N = 1130<br>Median age: 64 years (IQR: 60–68)	Robot-assisted laparoscopic radical prostatectomy	18 clinicopathological features: Age, race, BMI, PSA, % of scores positive, prostate biopsy Grade Group, tumor locality, prostate volume, largest tumor diameter, Extraprostatic Extension,	ML	**Classification** Naive Bayes (NB), RF, and SVM<br><br>**Regression** Logistic regression<br><br>**Nomograms** Kattan, CAPSURE, and Johns Hopkins	NR	70/30 train/validation split	Predictive Performance of Models at 1, 3, and 5 years:<br>NB (AUC=0.894, 0.876, 0.894)<br>RF (AUC= 0.846, 0.875, 0.888)<br>SVM (AUC=0.835, 0.850, 0.855)

					Seminal Vesicle Invasion, margins, nodal disease, perineural invasion, variant ductal histology, pathological grade group					
Luxton, J.J., et al. (2021) [51]	Prospective cohort pilot study<br><br>USA	Outcome prediction<br><br>Predict risk of radiation- induced late effects based on telomere length dynamics and chromosomal instability	N = 15 PCa patients	IMRT<br>All patients:<br>54 Gy<br>Patients receiving prostate fossa boost (N = 11):<br>70 Gy or 78 Gy	Telomere length dynamics and chromosomal instability	ML	**Regression**: XGBoost	Individual telomeres from the preIMRT non-irradiated time point were split into quartiles	5-fold-cross-validation, Leave-one-patient-out cross-validation	Mean Telomere Length Prediction:<br>$R^2 = 0.882$
Mostafaei, S., et al. (2023) [52]	Prospective cohort study<br><br>Iran, USA, Switzerland	Outcome prediction<br><br>Predict radiation-induced GI and urinary toxicities	N = 64 PCa patients<br><br>Mean age = 71 years<br><br>Median age = 72 years	IMRT:<br>52 patients (81%); Total dose = 70.2 Gy administered in 26 fractions.<br>3D Conformal Radiotherapy:<br>12 patients (19%); Total dose = 70 Gy delivered in 35 fractions.	CT radiomic features, dosimetric factors, and clinical factors (stage, Gleason score, age, PSA, prostate volume, planning target volume, conformity index, heterogeneity index, mean/min/max prostate dose, rectal and bladder dose volume including total	ML	**Classification:** RF, NN, and SVM	Elastic Net Logistic Regression	Repeated 5-fold cross-validation (100 repetitions)	Urinary Toxicity Prediction (AUC):<br>Clinical model: 0.67<br>Radiomics model: 0.71<br>Combined clinical–radiomics model: 0.77<br>GI Toxicity Prediction (AUC):<br>Clinical model: 0.66<br>Radiomics model: 0.71

					volume and wall volume and D5–D95 and V5–V75 for both total volume and wall volume)					Combined clinical–radiomics model: 0.65
Spratt, D., et al. (2023) [53]	Retrospective analysis of prospective RCTs<br><br>USA, Canada	Outcome prediction<br><br>Predict which patients with localized Pca will benefit from short-term androgen deprivation therapy when combined with RT	Development cohort:<br>N = 2024 patients<br>Median age = 71 (IQR: 65-74)<br>Validation cohort:<br>N = 1594 patients<br>Median age = 71 years (IQR: 66-74)	External-beam RT used<br><br>Hormone therapy (Androgen Deprivation Therapy):	Whole-image extraction + clinical data (PSA, Gleason score, primary/secondary Gleason pattern, T stage, age)	DL	Image-based DL via ResNet-50 with self supervised learning (MoCo-v2)	NR	External validation of independent cohort	RT + Androgen Deprivation Therapy: 15-year distant metastasis rate = 5.9%<br><br>RT alone:<br>15-year distant metastasis rate = 9.8%<br>Subdistribution Hazard Ratio = 0.64 (p = 0.01)
Abdollahi, H. et al., (2019) [45]	Prospective cohort study<br><br>Iran	Tumor Characterization & Outcome prediction<br><br>Predict early IMRT response in PCa patients, as well as Gleason Scores and PCa stages	N= 33 PCa patients<br>Mean = 70.5 ± NA yrs<br>(Range:51-82)<br>Median=73	IMRT: 70.2 Gy in 26 fractions	MRI radiomic features	ML	**Classification methods:** Linear SVM, Logistic Regression, Bernoulli NB, Stochastic Gradient Descent, K-nearest Neighbors (kNN), DT, RF, Adaptive Boosting (ADBO), and Gaussian NB	SelectKBest_chi squared, Variance Threshold, Select from Model, and Select Percentile	Ten-fold cross-validation	Gleason prediction w/ T2-weighted (T2W) radiomic model: AUC 0.739<br><br>Stage prediction w/ Apparent Diffusion Coefficient (ADC) radiomics: AUC 0.675

Massi, M., et al. (2020) [54]	Retrospective analysis of prospective cohort registry<br><br>Belgium, France, Germany, Italy, the Netherlands, Spain, UK, US	Outcome prediction<br><br>Predict risk of developing radiation- induced side effects based on SNPs associated with radiation toxicity in PCa patients	N=1,401 PCa patients<br><br>Age: NR	External beam RT	Single nucleotide polymorphisms (SNPs)	DL	**DL:** Deep Sparse AutoEncoders	Reconstruction error-based feature selection	Repeated subsampling (50 iterations)	AI confirmed that 20 out of 24 validated SNPs matched previous studies
Hassaninejad, H., et al. (2023) [55]	Prospective trial<br><br>Iran	Outcome prediction<br><br>Predicted rectal toxicity post RT	N=70 PCa<br>Mean= 71.05 ± NA years<br>(Range: 51–93)	Three-Dimensional Conformal RT: 45 Gy in 25 fractions	MRI-based radiomics	ML	**Classification:** RF,DT, Logistic Regression, kNN	LASSO	5-fold cross-validation	Best predictive model: kNN using MRI radiomic + dosimetric data<br>AUC: 0.86
McGarry, S., et al. (2018) [56]	Prospective cohort study<br><br>USA	Tumor characterisation<br><br>Characterized tumor tissue by quantifying epithelium and lumen density	N= 39<br>Mean=60 years old<br>Range= 45 to 72 years	Robotic-assisted radical prostatectomy	mpMRI radiomics	ML	**Regression:** Partial least squares (PLS)	Recursive greedy analysis, Correlation collinearity checks	Segmentation validation, Registration validation , Model stability validation , Predictive performance validation	Correlation between manual & computational segmentation for lumen (R = 0.99) & epithelium (R = 0.72; P < .001).<br>Dice coefficient: 94.5% overlap
Chrystall, D., et al. (2023) [57]	Retrospective analysis of prospectively collected data<br>Australia	Tumor characterization<br><br>Develop and evaluate a CNN-based fiducial marker tracking system for real time intrafractional motion	N = 29	Stereotactic Body Radiation Therapy using volumetric modulated arc therapy<br>36.25 Gy in 5 fractions	Whole-image extraction from stereotactic body radiation therapy	DL	**DL:** Binary Convolutional Neural Network (CNN)	NR	Independent test set validation	AUC: 0.9337

		monitoring in prostate SBRT								
Lee, S., et al. (2018) [58]	Retrospective analysis of prospectively collected data<br><br>USA	Outcome prediction<br><br>Predict patient-specific risk of late genitourinary toxicity after prostate RT using genome-wide SNPs	N = 324 patients	Volume modulated arc therapy:<br>76 Gy in 38 fractions	SNPs	ML	**Regression:** Preconditioned RF regression	Univariate filtering based on statistical significance	Two-phase validation:<br>1. Internal 5-fold cross-validation repeated 100 times<br>2. Independent hold-out test set (1/3 of patients, randomly split while matching event rates)	AUC for weak stream:<br>0.67 (95% CI: 0.64-70) in cross validation<br>0.70 (95% CI: 0.54-0.86) in hold out validation set
Rezaeijo, S. et al., (2021) [59]	RCT<br><br>Iran	Tumor characterization<br><br>Characterized tissue microarchitecture	N = 120<br>Age = 68±7	IMRT with simultaneous integrated boost:<br>80 Gy total	mpMRI radiomics	ML	**Clustering:** Hierarchical clustering, Agglomerative clustering	NR	Statistical validation:<br>t-tests, Analysis of Variance, Kruskal-Wallis	Planning Target Volume 3 Tumor Control Probability:<br>91.86% (Dose Painting) vs. 70.52% (IMRT)
Hu, L., et al. (2021) [60]	Prospective case control study<br><br>China	Outcome Prediction<br><br>PCa detection through zoomed vs full-FOV DWI radiomics with combined mpMRI clinical modeling	N = 136	NR	mpMRI radiomics + clinical risk factors (one was done with just radiomics, second part of the study was done with both); total PSA, free PSA, and ratio free/total PSA	ML	**Classification:** LASSO	LASSO (L1 regularization)	Holdout Validation	Independent Clinical risk factors model:<br>AUC = 0.74<br>mpMRI Model:<br>AUC = 0.92<br>Comprehensive model:<br>AUC = 0.93
Abdollahi, H. et al., (2018) [44]	Prospective cohort study	Outcome Prediction	N=33<br>Mean: 70.5<br>Median age: 73	IMRT: 70.2 Gy in 26 fractions.	Radiomic features from T2-Weighted	ML	**Classification**: Logistic regression	1. Robustness filtering<br>2. Filter-based	5-fold cross-validation	ADC radiomic models:<br>AUC mean =

	Iran	Early rectal toxicity prediction using pre/post-IMRT rectal wall radiomics	range (51-82)		Imaging (T2WI) and ADC MRI			feature selection		0.58 ± 0.034 for pre-IMRT<br>AUC mean = 0.56 ± 0.038 for post-IMRT
Abdollahi, H. et al., (2019) [46]	Prospective study<br><br>Iran	Outcome Prediction<br><br>Predicted urinary toxicity following IMRT using MRI texture features	N = 33<br>Mean age = 68.6 years<br>Range: 51–80 yrs	IMRT:<br>Patients were treated with 70.2 Gy in 26 fractions	MRI radiomics	ML	**Classification**: Logistic regression	Filter-based univariate ranking	AUC	Mean AUC = 0.65
Algohary, A., et al. (2022) [61]	Prospective collection of data, retrospective analysis<br><br>USA	Tumor characterization & outcome prediction<br><br>Predicted second year post-RT biopsy positivity	N = 25<br>Age: 68 ± 8 yrs	Lattice Extreme Ablative Dose boost RT	mpMRI-based radiomics	ML	**Regression:** Univariation logistic regression, Multivariate logistic regression	Correlation-based redundancy reduction	Leave-One-Out validation	*Note: Values are mean ± stdv<br><br>**Sequence: Diffusion Weighted Imaging (DWI); Feature: ADC (mm^2/sec)**<br>*12 months post*-RT*:*<br>Gross Tumour Volume: 1388.68±212.14<br>**Sequence: Dynamic Contrast-Enhanced (DCE); Feature: K^{trans} (min^{-1})**<br>*24 months post*-RT*:*<br>Gross Tumour Volume: 0.09±0.05

Section S3. The Risk Of Bias In Non-Randomized Studies of Interventions, Version 2 (ROBINS-I V2)

Defining Domains for ROBINS-I-V2

Note: 4 = Critical ROB; 3 = Serious ROB; 2 = Moderate ROB; 1 = Low ROB

Domain 1:

- Did the AI model include relevant clinical variables (e.g. age, PSA, comorbidities) as inputs (features) during model development/training?
  - 4 = no variables, 3 = one or two variables, 2 = three variables, 1 = four or more variables
- Was there imbalance in baseline characteristics (patient traits before intervention, such as age, comorbidities, PSA, Gleason score, T stage, treatment type) between groups (e.g. training vs. test sets or cases vs. controls)? Was the imbalance adjusted for in analysis?
  - If there was imbalance, but it was adjusted for appropriately OR there was no imbalance, then the rating is 1.
  - If there was imbalance that was not adjusted for, then give a rating of 2-4 based on the severity of imbalance.

Domain 2:

- Was the AI model applied before outcome occurrence?
  - 4 = uses only post-treatment data as input, 2-3 = mixed, 1 = pretreatment data only
- Was the input data (e.g., images) labeled (segmentations, region of interest) without knowledge of the patient's outcome at the time?
  - 4 = radiologists were not blinded, 3-2 = some blinding, 1 = blinded or automated labelling

Domain 3:

- Were patients <u>included</u> based on post-intervention information?
  - 4 = selection was based on outcome, 1 = consecutive or eligible sampling

Domain 4:

- Were the AI predictions applied uniformly?
  - 4 = severe variation in preprocessing/how the AI was applied, 3 = serious variation, 2 = moderate variation, 1 = no variation in how AI was applied

Domain 5:

- Was there missing data for any of the patients' outcome, input, or confounder data?
  - 4 = if a lot of missing data, 3-2 = some, 1 = no missing data
- If there was missing data, was it handled via imputation or by only analysing patients with full data (complete-case analysis)?
  - 4-3 = data was ignored, the study didn't specify what happened to the data, or biased imputation.
  - 2 = Explained <u>why</u> they only analysed the patients they did
  - 1 = unbiased imputation

Domain 6:

- Was the outcome defined with validated/standardised criteria?
  - 1 = yes, 2-3 = criteria is not so rigorous, 4 = no
- Was the physician who assessed the outcome blinded to what the AI predicted or what the imaging features looked like?
  - Yes = low risk (1-2), no = high risk (3-4)

Domain 7:

- Was there a published protocol, registry, or plan describing which outcomes and models would be reported?
  - Yes = low risk (1-2), no = high risk (3-4)
- Were multiple models tested but only reported the one that performs the best?
  - Yes = high risk (3-4).
  - No OR rationale is provided for why only one model is reported = low risk (1-2).

Table S3. Raw ROB table (using ROBINS-I V2)
Note: 4 = Critical ROB; 3 = Serious ROB; 2 = Moderate ROB; 1 = Low ROB

Paper	Domain 1	Domain 2	Domain 3	Domain 4	Domain 5	Domain 6	Domain 7	Total score
Yang, Z., et al (2023) [47]	4<br>2	1<br>1	1	1	2<br>2	1<br>3	3<br>1	22/48
Grajales, D., et al (2022) [23]	3<br>2	1<br>1	1	1	1<br>N/A	1<br>3	2<br>2	18/48
Marturano, F., et al. (2023) [48]	1<br>1	1<br>3	1	1	2<br>1	1<br>3	3<br>2	20/48
Sun, Y., et al. (2023) [49]	1<br>2	1<br>3	1	1	3<br>3	1<br>3	3<br>1	23/48
Tan, Y., et al. (2021) [50]	1<br>1	1<br>4	1	1	2<br>1	1<br>4	4<br>1	23/48
Luxton, J.J., et al. (2021) [51]	4<br>4	1<br>1	1	1	2<br>2	4<br>4	4<br>1	29/48
Mostafaei, S., et al. (2023) [52]	1<br>1	1<br>1	1	1	2<br>2	1<br>2	4<br>4	21/48
Spratt, D., et al. (2023) [53]	2<br>2	1<br>2	1	1	2<br>2	1<br>2	2<br>2	20/48
Abdollahi, H. et al., (2019) [45]	4<br>2	3<br>4	1	1	2<br>1	2<br>4	4<br>2	30/48

Massi, M., et al. (2020) [54]	3<br>4	1<br>1	1	1	2<br>3	1<br>1	4<br>1	23/48
Hassaninejad, H., et al. (2023) [55]	4<br>2	1<br>1	1	1	1<br>1	1<br>N/A	3<br>1	17/48
McGarry, S., et al. (2018) [56]	2<br>1	1<br>4	1	1	1<br>1	1<br>1	3<br>1	18/48
Chrystall, D., et al. (2023) [57]	4<br>2	1<br>1	1	1	1<br>1	4<br>1	4<br>1	22/48
Lee, S., et al. (2018) [58]	2<br>1	1<br>1	1	1	2<br>2	1<br>2	3<br>3	22/48
Rezaeijo, S. et al., (2021) [59]	4<br>3	1<br>3	1	1	3<br>3	4<br>2	3<br>1	29/48
Hu, L., et al. (2021) [60]	2<br>1	1<br>4	1	1	1<br>2	2<br>4	4<br>1	24/48
Abdollahi, H. et al., (2018) [44]	4<br>2	2<br>3	1	1	1<br>1	1<br>3	4<br>2	25/48
Abdollahi, H. et al., (2019) [46]	4<br>1	3<br>4	1	1	1<br>1	2<br>4	4<br>4	30/48
Algohary, A., et al. (2022) [61]	4<br>1	3<br>4	2	1	2<br>2	1<br>4	4<br>4	32/48

Section S4. Study Categorization

Table S4. Categorization table of the 19 eligible studies

Category	Specific Papers	Total Number
# of studies that are from X country	Australia: Chrystall 2023<br>Canada: Grajales 2022<br>China: Sun 2023, Hu 2021<br>Iran: Mostafaei 2020, Abdollahi 2019, Hassaninejad 2023, Rezaeijo 2021, Abdollahi 2018, Abdollahi 2019<br>Italy: Marturano 2023<br>Singapore: Tan 2021<br>United Kingdom: Yang 2023<br>United States: Luxton 2021, McGarry 2018, Lee 2018, Algohary 2022<br>Massi 2020 – affiliated with 8 countries:<br>Italy, France, Germany, United Kingdom, United States, Netherlands, Canada, Spain<br>Spratt 2023 – affiliated with 2 countries:<br>United States, Canada	Australia: 1<br>Canada: 1<br>China: 2<br>Iran: 6<br>Italy: 1<br>Singapore: 1<br>United Kingdom: 1<br>United States: 4<br><br>(Massi: 8 countries<br>Spratt: 2 countries)
# of studies that are published in X year	2024: Sun 2023<br>2023: Yang, Marturano, Spratt, Hassaninejad, Chrystall<br>2022: Grajales, Tan 2021, Algohary<br>2021: Luxton, Rezaeijo, Hu<br>2020: Mostafaei, Massi<br>2019: Abdollahi, Abdollahi<br>2018: McGarry, Lee, Abdollahi	2024: 1<br>2023: 5<br>2022: 3<br>2021: 3<br>2020: 2<br>2019: 2<br>2018: 3
# of studies that are ML	Yang 2023, Grajales 2022, Marturano 2023, Sun 2023, Tan 2021, Luxton 202, Mostafaei 2020, Abdollahi 2019, Abdollahi 2019, Hassaninejad 2023, McGarry 2018, Lee 2018, Rezaeijo 2021, Hu 2021, Abdollahi 2018, Algohary 2022	16
# of studies that are DL	Spratt 2023, Massi 2020 , Chrystall 2023	3
# of studies that use radiomics only as the input	Rezaeijo 2021, Abdollahi 2019, Hassaninejad 2023, Algohary 2022, Abdollahi 2018, Abdollahi 2019	6
# of studies that use radiomics + other	Yang 2023: Radiomics + dosimetrics<br>Grajales 2022: Radiomics+ biomolecular features from RS<br>Marturano 2023: Radiomics + clinical data<br>Sun 2023: Radiomics + risk factors<br>Mostafaei 2020: Radiomic features + dosimetric factors + clinical factors<br>McGarry 2018: Radiomics + digitized histopathology<br>Hu 2021: Radiomics + clinical risk factors	7

# of studies that use another input instead of radiomics	Tan 2021: 18 clinicopathological features were extracted from each patient<br>Luxton 2021: Telomere length dynamics and chromosomal instability<br>Massi 2020 & Lee 2018: SNPs<br>Chrystall 2023: Whole-image extraction<br>Spratt 2023: Whole-image extraction + clinical data	6
# of studies that are about tumor characterization	Grajales 2022, Sun 2023, McGarry 2018, Chrystall 2023, Rezaeijo 2021	5
# of studies that are about outcome prediction	Abdollahi 2019	2
# of studies that are about both	Yang 2023, Marturano 2023, Tan 2021, Luxton 2021, Mostafaei 2020, Spratt 2023, Massi 2020, Hassaninejad 2023, Lee 2018 , Hu 2021, Abdollahi 2018, Abdollahi 2019 , Algohary 2022	12
# of studies that are RCT	Rezaeijo 2021	1
# of studies that are prospective studies (prospective in data collection AND analysis)	Grajales 2022, Marturano 2023, Sun 2023, Luxton 2021, Mostafaei 2020, Abdollahi 2019, Hassaninejad 2023, McGarry 2018, Hu 2021, Abdollahi 2018, Abdollahi 2019	11
Studies that are prospective in data collection but retrospective in analysis	Yang 2023, Tan 2021, Spratt 2023, Massi 2020, Chrystall 2023, Lee 2018, Algohary 2022	7
# of studies that use X imaging modality as the input for the AI algorithm	CT: Yang 2023, Marturano 2023, Mostafaei 2020, Hassaninejad 2023, Abdollahi 2019<br>PET: Marturano 2023<br>In-situ Raman Spectroscopy: Grajales 2022<br>mpMRI: Grajales 2022, McGarry 2018, Rezaeijo 2021, Hu 2021, Algohary 2022<br>MRI: Abdollahi 2019, Hassaninejad 2023, Abdollahi 2018, Abdollahi 2019<br>Conventional TRUS (B-mode) and CEUS: Sun 2023<br>Megavoltage cine-imaging: Chrystall 2023	Total: 18<br><br>CT: 5<br>PET: 1<br>In-situ Raman spectroscopy: 1<br>mpMRI: 5<br>MRI: 4<br>Conventional TRUS (B-mode) and CEUS: 1<br>Megavoltage cine-imaging: 1

# of studies that use classification algorithms	SVM: Yang 2023, Grajales 2022, Tan 2021, Mostafaei 2020, Abdollahi 2019<br>LightGBM: Yang 2023<br>NN: Yang 2023, Mostafaei 2020<br>LASSO logistic regression: Marturano 2023, Sun 2023, Hu 2021<br>Elastic Net penalised logistic regression: Mostafaei 2020<br>Logistic regression: Tan 2021, Abdollahi 2019, Hassaninejad 2023, Abdollahi 2018, Abdollahi 2019<br>Univariate logistic regression: Algohary 2022<br>Multivariate logistic regression: Algohary 2022<br>NB: Tan 2021, Abdollahi 2019<br>RF: Tan 2021, Mostafaei 2020, Abdollahi 2019, Hassaninejad 2023<br>CNN: Spratt 2023, Chrystall 2023<br>kNN: Abdollahi 2019, Hassaninejad 2023<br>DT: Abdollahi 2019, Hassaninejad 2023<br>ADBO: Abdollahi 2019	Total: 17<br><br>SVM: 5<br>LightGBM: 1<br>NN: 2<br>LASSO logistic regression: 3<br>Elastic Net penalised Logistic regression: 1<br>Logistic regression: 5<br>Univariate logistic regression: 1<br>Multivariate logistic regression: 1<br>NB: 2<br>RF: 4<br>CNN: 2<br>kNN: 2<br>DT: 2<br>ADBO: 1
# of studies that use regression algorithms	Linear regression: Luxton 2021<br>PLS regression: McGarry 2018<br>Preconditioned RF regression: Lee 2018<br>XGBoost: Luxton 2021	Total: 3<br><br>Linear regression: 1<br>PLS regression: 1<br>Preconditioned RF regression: 1<br>XGBoost: 1
# of studies that use clustering algorithms	Hierarchical clustering: Rezaeijo 2021<br>Agglomerative clustering: Rezaeijo 2021	Total: 1<br><br>Hierarchical clustering: 1<br>Agglomerative clustering: 1
# of studies that use another algorithm	Deep Sparse Auto Encoders: Massi 2020; Unsupervised anomaly detection and feature effect estimation	Total: 1<br><br>Deep Sparse Auto Encoders: 1
